# Poor Dietary Habits in Bullied Adolescents: The Moderating Effects of Diet on Depression

**DOI:** 10.3390/ijerph15081569

**Published:** 2018-07-24

**Authors:** Natalia Albaladejo-Blázquez, Rosario Ferrer-Cascales, Nicolás Ruiz-Robledillo, Miriam Sánchez-Sansegundo, Violeta Clement-Carbonell, Ana Zaragoza-Martí

**Affiliations:** 1 Department of Health Psychology, Faculty of Health Science, University of Alicante, 03690 Alicante, Spain; natalia.albaladejo@ua.es (N.A.-B.); nicolas.ruiz@ua.es (N.R.-R.); miriam.sanchez@ua.es (M.S.-S.); violeta.clement@ua.es (V.C.-C.); 2 Department of Nursing, Faculty of Health Science, University of Alicante, 03690 Alicante, Spain; ana.zaragoza@ua.es

**Keywords:** bullying, diet, depression, adolescents

## Abstract

The prevalence of bullying has increased dramatically during recent years, with numerous negative consequences for the health and quality of life of bullied adolescents. Although negative psychological consequences of this type of situation have been widely investigated, no previous research has evaluated the effects of bullying victimization on dietary habits, and its relationship with psychological outcomes, such as depression. For this reason, the main aim of the present study was to evaluate the association between bullying, dietary habits, and depression in a sample of 527 Spanish adolescents. The results obtained showed that being bullied was correlated negatively with healthy dietary habits and positively with depression. Moderation analysis revealed dietary habits as moderator of the association between bullying and depression. These results underline the relevance of diet in the phenomenon of bullying, especially in victims, as could be related to the high levels of depression characteristic of this population. The inclusion of nutritional education in intervention programs oriented to victims of bullying might significantly improve their efficacy, reducing depression levels.

## 1. Introduction

Bullying victimization is defined as the repeated occurrence of abuse between people from the same age group where an imbalance of power makes it difficult for the victims to defend themselves [1]. This complex phenomenon has been increasing dramatically across the world over recent years [2] with an estimated prevalence of 36% according to a recent meta-analysis [3]. Bullying victimization has shown to have severe negative consequences for the health and quality of life of bullied adolescents, such as higher levels of depression, anxiety, somatic symptoms, and suicidal ideation [4,5,6]. In line with this, a recent study conducted with 6667 students concluded that being bullied has a significant negative impact on physical, psychological, and social domains of quality of life [7,8].

Several studies have confirmed that, as expected, victims of bullying show higher levels of depression than nonbullied adolescents [9,10,11,12], this being one of the most widely studied consequences of bullying victimization in previous research [9,10,11]. Further, it has been demonstrated that bullying victimization is followed by depression, reaching clinical levels in some cases [9]. This issue is especially worrisome considering that depression has shown to be a significant mediator in the association between bullying victimization and nonsuicidal self-injury [11] and a moderator of suicide risk [12]. In fact, bullying victims are at a higher risk of self-harm, suicidal ideation, suicidal planning, and suicide attempts than nonbullied adolescents [4].

While the phenomenon of bullying has been widely analyzed in previous research, revealing a severe health risk, the majority of studies to date have only evaluated emotional and psychosocial consequences of being bullied, but few studies have explored the relationship between bullying and dietary habits [13,14]. In this sense, it has recently been found that adolescents who are victims of bullying and cyberbullying frequently report skipping breakfast [13,14]. Taking into account that bullying entails high levels of psychological dysfunction and stress, victims are at increased risk of appetite deregulation and, hence, skipping meals [13,14]. To our knowledge, no research has analyzed the possible deterioration of dietary patterns in bullying victims, and its association with mood disorders, such as depression.

Therefore, the aims of the present study were to evaluate the relationship between bullying victimization, dietary habits, and depression in a sample of Spanish adolescents, and to analyze differences in dietary habits between high- and low-victimised adolescents. Furthermore, the study aimed to identify the prediction ability of bullying and dietary habits on depression and to evaluate the possible moderation effects of dietary habits in the relationship between bullying and depression. Based on previous research, we hypothesized that bullying victimization would be negatively related to healthy dietary habits [8,13,14] and positively related to depression [9,11]. Although previous research had not evaluated the possible prediction and moderation effects of diet in this population, we expected to find that bullying victims with high adherence to a healthy dietary habit would show lower rates of depression [15,16].

## 2. Materials and Methods

### 2.1. Procedure

The present study is a part of a large-scale study on the Mediterranean Diet (MD), wellbeing, and victimization carried out in schools in the Mediterranean city of Alicante (Spain). The study was approved by the Ethics Committee of the University of Alicante (UA-2015-10-13), and parents provided consent to the participation of their children prior to data collection. The participants were 527 high school students (54.5% females; 45.5% males) ranging in age from 12 to 17 years (M = 14.43, SD = 1.52) randomly selected from 5 public high schools in Alicante (Spain). Students who assented to participate anonymously completed a battery of questionnaires in a paper-pencil format. The distribution and completion of questionnaires was overseen by research assistants during the second and third trimesters of the 2015/2016 academic year and the process took 60 to 70 min.

Inclusion criteria for the students were: (1) being present in the classroom on the day of the survey, (2) being able to read and complete the questionnaires on their own, and (3) presenting an informed consent form signed by their parents allowing participation. Participants were only retained in the final sample if they had completed all questionnaires concerning the primary dependent measures of bullying, dietary habits, and depression.

### 2.2. Measures

#### 2.2.1. Bullying Victimization

For the analysis of bullying victimization, the subscale “victimization received” from the Multimodal Questionnaire of School Interaction (MQSI) [17] was employed. This factor comprises 10 items, rated on a 4-point Likert scale, and evaluates the frequency of victimization received by a bullied child or victim. Questions included in the subscale refer to victimization from verbal (e.g., “they have insulted me”), social (e.g., “they have ignored me”), and physical (e.g., “they hit me”) bully behaviors. This instrument has demonstrated adequate internal consistency among adolescents [17]. Cronbach’s alpha values for the subscale of victimization received was 0.86.

#### 2.2.2. Dietary Habits

Dietary habits were evaluated by The MD Quality Index for children and teenagers (KIDMED) [18,19]. It consists of 16 questions rated on a scale from 0 to 12 and can be self-administered or administered by an interviewer following a protocol. Dietary items included in the questionnaire evaluate the characteristic dietary pattern of the MD, a recognized healthy dietary pattern, including items referring to the frequency of the consumption of fruits and vegetables, pulses, cereals, dairy products and fish, the use of olive oil in cooked meals at home, and questions regarding breakfast consumption and the characteristics of the breakfast usually consumed (if it includes dairy products, baked goods, or pastries). The questionnaire also included two items related to the frequency of sweets and fast food consumption. The total score on the questionnaire is classified into three levels: ≥8, indicating an “optimal” Mediterranean diet, 4–7, that improvement is needed to adjust intake to Mediterranean patterns, and ≤3, very low diet quality. This questionnaire has demonstrated adequate test-retest reliability [20] and construct validity [21] in previous research. In the present study, Cronbach’s alpha for the total scale was 0.71.

#### 2.2.3. Depression

The Short Web-Based version of the Center for Epidemiological Studies Depression Scale (CES-D) [22] is composed of 7 items and can be used to evaluate the presence of depressive symptomatology in children and adolescents. It covers the following domains: depressed affect, positive affect, somatic and retarded activity, and interpersonal difficulties. Responses are rated on a 4-point Likert scale. The total score, employed as a general measure of depressive mood, was used in this study. The CES-D has shown adequate psychometric properties to estimate depression in several samples [22,23]. The Cronbach’s alpha value in the current sample for this scale was 0.70.

### 2.3. Data Analysis

Pearson’s correlations were used to analyze the relationships between bullying, dietary habits, and depression. Participants were divided into two groups (high-victimized* n* = 235 (44.6%) and low-victimized *n* = 292 (55.4%) adolescents) based on their total scores on the bullying victimization scale by using a cluster analysis. Chi-square analyses were performed to evaluate differences between high- and low-victimized adolescents’ dietary habits. Hierarchical linear regression analysis was used to determine the predictive value of bullying and dietary habits on depression. To test the moderation effect of diet on the relationship between bullying and depression, the macro PROCESS by Hayes was employed [24]. All statistical analyses were performed using SPSS (International Business Machines Corporation (IBM), Armonk, NY, USA), Statistics for Windows, Version 24.0, considering any *p* < 0.05 as significant.

## 3. Results

### 3.1. Relationship between Bullying, Dietary Habits, and Depression

Pearson’s correlations between bullying, dietary habits, and depression are presented in Table 1. Bullying was negatively associated with all of the evaluated dietary habits, except with fast food, sweets, commercially baked goods, and breakfast consumption. The same pattern of correlations were found for depression, except in the case of fast food and sweet consumption, in which depression showed a significant positive association (Table 1).

### 3.2. Differences between High and Low Victimised Adolescents in Dietary Habits

To evaluate differences in dietary habits depending on bullying scores, differences between high- and low-victimized adolescents were analyzed. Significant differences were found in all of the evaluated dietary habits (*p *< 0.01), except in the case of breakfast and sweets consumption. In all cases, high-victimized adolescents exhibit poorer dietary habits in comparison to low-victimized adolescents (Table 2).

### 3.3. Prediction Ability of Bullying and Dietary Habits on Depression

In order to evaluate the ability of bullying and dietary habits to predict depression, a hierarchical regression model was constructed. For controlling the possible confounding effects of age and sex, these two factors were included in the first step. Bullying was included in the second step, and dietary habits in the third. When age and sex were introduced in the first step, neither were significant predictors. In the second step, including bullying in the model, this factor was found to be significant. In the third step, in which dietary habits were included, bullying remained significant and all of the evaluated dietary habits except the use of olive oil were significant. In this final step, age and sex were not significant predictors of depression (Table 3).

### 3.4. Moderation Effects of Dietary Habits on the Relationship between Bullying and Depression.

**Figure 1:**
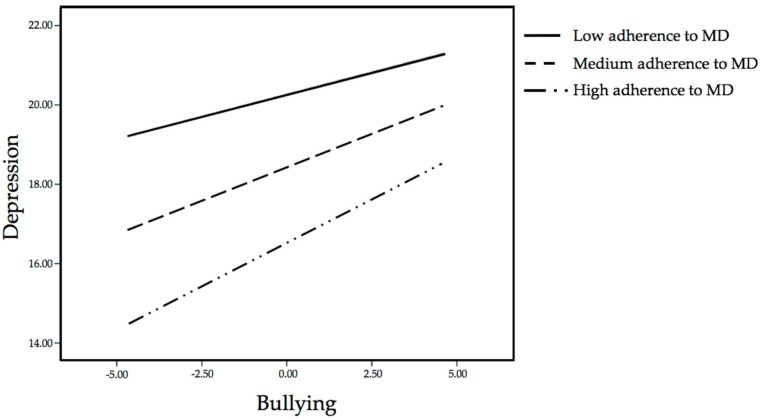
Graphical representation of the moderation of dietary habits (adherence to MD) on the association between bullying and depression.

The hypothesized model suggested a significant interaction effect of bullying and dietary habits (level of adherence to MD) on depression (*p* = 0.00001). Taking into account that the lower and upper limits of the 95% confidence interval for the interaction did not cross zero, the significant effects of the interaction between both variables could be corroborated (Table 4). Hence, the model was significant R^2^ = 0.81, MSE = 1.99, F(3, 523) = 764.07, *p* = 0.00001. The moderation effect is plotted in Figure 1. According to the Johnson–Neyman technique, the relationship between bullying and depression was moderated by dietary habits at all levels. For that reason, a slope analysis was conducted to observe the moderation effects of dietary habits, depending on the level of adherence to MD (high, medium and low). Simple slopes analyses showed that the relationship between bullying and depression was significant at one standard deviation below the mean adherence to MD score (b = 0.223, SE = 0.047, t = 4.68, *p* = 0.00001, 95% CI: 0.129, 0.317), at the mean adherence to MD score (b = 0.332, SE = 0.067, t = 4.93, *p* = 0.00001, 95% CI: 0.200, 0.464), and at one standard deviation above the mean adherence to MD (b = 0.441, SE = 0.089, t = 4.94, *p* = 0.0001, 95% CI: 0.266, 0.616). As can be observed, there is an interaction effect between bullying and adherence to MD, showing those adolescents with high adherence to MD have lower rates of depression in comparison to adolescents with medium and low adherence to MD (Figure 1).

## 4. Discussion

In this study, we have examined (i) the relationship between bullying victimization, dietary habits, and depression; (ii) differences in dietary habits between high- and low-victimized adolescents; (iii) the prediction ability of bullying and dietary habits on depression; and (iv) the moderation effects of diet on depression. Our results show that bullying victimization is significantly related to poor dietary habits together with higher levels of depression in Spanish adolescents. Hence, high-victimized adolescents showed poor dietary habits compared to low-victimized ones. Regression and moderation analyses exhibited a significant effect of diet on depression.

Regarding the association between bullying and dietary habits, results have demonstrated a significant association between bullying victimization and poor dietary habits corroborated by the analyzed differences between high- and low-victimized adolescents. It has been previously demonstrated that bullying as a chronic stress situation is related to significant negative changes in lifestyle in adolescents, such as a decline in diet quality [25]. In this regard, chronically-stressed adolescents are less likely to have a healthy lifestyle in which dietary habits play a main role [25,26]. Further, it has previously been shown that highly-stressed children and adolescents tend to develop an unhealthy dietary pattern, characterized by a higher intake of sweets and fatty foods than fruit and vegetables [27,28] This could be explained by the high content of fats, energy, and sugar of these kinds of foodstuff; but, it could be mediated more by psychological perception and preferences than by nutritional composition [28]. Hence, based on the theory of “comfort foods”, the consumption of these kinds of foodstuff, rich in sugar and fats, could represent an escape coping strategy due to the emotional component of eating and the rewarding effects of this type of food, reducing perceived stress [28]. Recent research indicates that leptin may play a role as a mediator between psychosocial stress and emotional eating characteristic of stressed individuals [29]. Specifically, chronic stress induces high cortisol levels and, in turn, high leptin concentrations due to resistance mechanisms associated with greater intake of comfort food [29]. This fact could explain, in part, the poor dietary habits of bullied adolescents as a chronically stressed population. All of this could determine one of the many mechanisms studied relating bullying and a negative mood state, such as depression.

Remembering that the definition of bullying victimization excludes occasional or minor incidents (it being necessary that the behavior is repeated over time), this situation can be characterized as a form of chronic stress [30]. Chronic stress, defined as a situation in which the body faces numerous challenges every day, has shown to be one of the most dangerous situations for the maintenance of homeostasis, due to allostatic overload [6,31]. In particular, daily challenges from bullying pose a threat to allostasis (a set of processes that allow organisms to maintain homeostasis while adapting to the demands of the environment) [32]. Disturbances in homeostasis result in allostatic load, a state in which physiological systems are not able to adapt to environmental changes, with severe consequences for health and well-being [31,33]. Chronic overactivation of psychobiological stress mechanisms, such as the release of catecholamines following activation of the sympathetic-adrenal-medullary axis and the secretion of glucocorticoids following activation of the hypothalamic-pituitary-adrenal axis, leaves the body at high risk of developing stress-related diseases [33] such as depression. However, previous research has demonstrated that several variables could buffer these negative consequences of chronic stress on mood, demonstrating that diet is closely related to allostatic load [34,35]. In this regard, recent reviews and meta-analyses have demonstrated that a high-quality diet is associated with a lower risk of depressive symptoms [36,37,38]. There are a number of biological pathways that could explain that adolescents who maintain a high-quality diet could buffer the consequences of derived stress from bullying, reducing the risk of suffer depressive symptoms. The dietary intake of folate, zinc, and magnesium typical of a healthy dietary pattern, such as the MD, or the reduction of systemic inflammation, oxidative stress, and adaptive brain development could be on the basis of the protective effect of adequate dietary habits in chronically-stressed adolescents [36,37,38,39,40]. These mechanisms could explain the obtained results in the present study, in which individuals with healthy dietary habits suffer from lower levels of depression, although they also suffer from high levels of bullying.

The present results show the necessity of the implementation of effective antibullying strategies in schools, considering the severity of the consequences derived from this phenomenon. A recent systematic review has demonstrated the differential effectiveness of specific antibullying interventions for reducing bully behaviors at schools [41]. Although several types of interventions demonstrated a positive effect increasing positive school climate and reducing bullying at schools, multicomponent or whole school interventions were the most effective type of antibullying programs [41,42]. These kinds of interventions combine classroom rules, lectures addressing bullying, activities with bullies and victims, parents involvement, increased supervision, disciplinary methods, training of teachers, and technological resources [41]. According to previous research, these interventions, which include a wide variety of activities and skills development, have exhibited higher effectiveness compared to those delivered through classroom curricula or social skills training alone [41,42]. In the schools participating in the present study, a bullying intervention program based on emotional education is currently being implemented. This type of intervention includes activities such as contents exposition, group discussions, role-playing, cooperative learning, and videos, the main aim being the promotion of emotional intelligence in students through the increase of emotional awareness and regulation. Future studies should examine the role of emotional competences as antibullying strategies in school.

Although the present study represents an advance in our understanding of dietary habits in bullied adolescents, some limitations should be taken into account. The cross-sectional nature of the study is a methodological limitation; specifically, the nonexperimental design means that causality cannot be established. In relation to this, longitudinal studies are needed to explore how dietary habits could protect bullied adolescents from the negative consequences of bullying on health, reinforcing the effectiveness of traditional intervention programs through the inclusion of dietary education. On the other hand, it should be taken into account that the present study is the first to analyze the relationship between bullying victimization, specific dietary habits, and depression.

## 5. Conclusions

Given that the results obtained show a low adherence to healthy dietary habits in bullied adolescents, it is important to develop intervention programs oriented to the promotion of healthy eating habits in this population. Furthermore, interventions oriented to emotional regulation and coping with stress in an adaptive manner in this population could significantly benefit the health of bullying victims through reducing emotional eating. The present study shows the importance of nutritional variables as factors that may be protective of health in bullying victims. The inclusion of nutritional education in intervention programs should be considered by clinicians and other professionals involved in treating and preventing the negative consequences of being bullied. Future studies should investigate the effectiveness of dietary interventions in the improvement of dietary habits and the reduction of depression in bullying victims.

## Figures and Tables

**Table 1:** Pattern of Pearson’s correlations between bullying, depression, and dietary habits (* *p* < 0.05; ** *p* < 0.001).

	Bullying	Depression
Fruit or fruit juice daily	−0.723 **	−0.594 **
Second serving of fruit daily	−0.541 **	−0.547 **
Fresh or cooked vegetables daily	−0.478 **	−0.477 **
Fresh or cooked vegetables >1/day	−0.504 **	−0.542 **
Regular fish consumption (at least 2–3/week)	−0.476 **	−0.434 **
Fast-food (hamburger) restaurant >1 week	0.082	0.104 *
Pulses >1/week	−0.279 **	−0.320 **
Pasta or rice almost daily (≥5/week)	−0.289 **	−0.287 **
Cereal or cereal product for breakfast	−0.471 **	−0.452 **
Regular nut consumption (at least 2–3/week)	−0.269 **	−0.251 **
Use of olive oil at home	−0.377 **	−0.260 **
No breakfast	0.123 **	0.076
Dairy product for breakfast	−0.628 **	−0.575 **
Commercially baked goods or pastries for breakfast	0.014	−0.052
Two yogurts and/or 40 g cheese daily	−0.347 **	−0.349 **
Sweets and candy several times a day	0.072	0.095 *

**Table 2:** Differences between high- and low-victimized adolescents in dietary habits.

		High Victimization *n* = 235	Low Victimization *n* = 292	
Fruit or fruit juice daily	No	105	6	χ² = 142.297, *p* = 0.0001
	Yes	130	286	
Second serving of fruit daily	No	182	78	χ² = 134.086, *p* = 0.0001
	Yes	53	214	
Fresh or cooked vegetables daily	No	133	40	χ² = 108.660, *p* = 0.0001
	Yes	102	252	
Fresh or cooked vegetables >1/day	No	206	100	χ² = 152.560, *p* = 0.0001
	Yes	29	192	
Regular fish consumption (at least 2–3/week)	No	144	56	χ² = 97.998, *p* = 0.0001
	Yes	91	236	
Fast food (hamburger) restaurant >1 week	No	152	216	χ² = 5.336, *p* = 0.021
	Yes	83	76	
Pulses >1/week	No	118	63	χ² = 47.356, *p* = 0.0001
	Yes	117	229	
Pasta or rice almost daily (≥5/week)	No	133	83	χ² = 42.722, *p* = 0.0001
	Yes	102	209	
Cereal or cereal product for breakfast	No	147	63	χ² = 91.218, *p* = 0.0001
	Yes	88	229	
Regular nut consumption (at least 2–3/week)	No	147	105	χ² = 36.907, *p* = 0.0001
	Yes	88	187	
Use of olive oil at home	No	29	5	χ² = 24.369, *p* = 0.0001
	Yes	206	287	
No breakfast	No	179	208	χ² = 1.627, *p* = 0.202
	Yes	56	84	
Dairy product for breakfast	No	139	36	χ² = 128.691, *p* = 0.0001
	Yes	96	256	
Commercially baked goods or pastries for breakfast	No	152	216	χ² = 5.336, *p* = 0.021
	Yes	83	76	
Two yogurts and/or 40 g cheese daily	No	185	128	χ² = 65.713, *p* = 0.0001
	Yes	50	164	
Sweets and candy several times a day	No	194	257	χ² = 3.146, *p* = 0.076
	Yes	41	35	
Low adherence to Mediterranean Diet (MD)		103	0	χ² = 350.725, *p* = 0.0001
Medium adherence to MD		132	65	
High Adherence to MD		0	227	

**Table 3:** Predictive values of age, sex, bullying, and dietary habits in depression (* *p* < 0.05; ** *p* < 0.001).

Model 1	β	R^2^	ΔR^2^
Age	−0.012		
Sex	−0.061		
F(2, 526) = 0.972, *p* = 0.379		0.000	0.004
Model 2	β	R^2^	ΔR^2^
Age	0.035		
Sex	0.001		
Bullying	0.834 **		
F(3, 526) = 394.983, *p* = 0.0001		0.692	0.690 **
Model 3	β	R^2^	ΔR^2^
Age	0.004		
Sex	0.001		
Bullying	0.118 *		
Fruit or fruit juice daily	−0.106 **		
Second serving of fruit daily	−0.148 **		
Fresh or cooked vegetables daily	−0.126 **		
Fresh or cooked vegetables >1/day	−0.173 **		
Regular fish consumption (at least 2–3/week)	−0.157 **		
Fast food (hamburger) restaurant >1 week	0.115 **		
Pulses >1/week	−0.153 **		
Pasta or rice almost daily (≥5/week)	−0.142 **		
Cereal or cereal product for breakfast	−0.145 **		
Regular nut consumption (at least 2–3/week)	−0.119 **		
Use of olive oil at home	−0.036		
No breakfast	0.132 **		
Dairy product for breakfast	−0.140 **		
Commercially baked goods or pastries for breakfast	0.143 **		
Two yogurts and/or 40 g cheese daily	−0.150 **		
Sweets and candy several times a day	0.095 **		
F(19, 526) = 113.735, *p* = 0.0001		0.803	0.116 **

**Table 4:** Moderation of the relationship between bullying and depression by dietary habits (Adherence to MD).

	B	SE	t	*p*	95% CI
Bullying	0.332	0.067	4.930	0.00001	[0.200, 0.464]
Dietary habits (Adherence to MD)	−0.689	0.084	−8.151	0.00001	[−0.855, −0.523]
Bullying x Dietary habits (Adherence to MD)	0.040	0.008	4.546	0.00001	[0.022, 0.057]
